# The Effect of Active and Passive Maternal Smoking During Pregnancy on the Uterine Artery Blood Flow and Obstetric Outcomes: A Prospective Study

**DOI:** 10.7759/cureus.35270

**Published:** 2023-02-21

**Authors:** Sema Baki Yıldırım, Kıymet İclal Ayaydın Yılmaz, Cavidan Gulerman

**Affiliations:** 1 Gynecology and Obstetrics, Giresun University Faculty of Medicine, Gynecology and Pediatrics Training and Research Hospital, Giresun, TUR; 2 Department of Obstetrics and Gynecology, Zekai Tahir Burak Women Health Care Education and Research Hospital, Ankara, TUR

**Keywords:** uterine artery doppler, umbilical artery doppler, obstetric outcomes, neonatal complications, maternal smoking

## Abstract

Background and Aim: Maternal smoking is associated with an increased risk of obstetric and neonatal complications during pregnancy. We aimed to investigate the effects of active and passive smoking on fetal-maternal blood flow and fetal complications in mid-trimester pregnant women.

Methods: This prospective study was conducted at Zekai Tahir Burak Women’s Research and Training Hospital and included women who were 20 to 22 weeks old, with no fetal anomalies, and with a singleton pregnancy. The data used in the analysis were obtained from a total of 168 pregnant women (50 smokers, 50 passive smokers, and 68 non-smokers). Starting from their 20^th^ week of pregnancy, the women were examined at least once in each trimester. Fetal and maternal pregnancy results were evaluated. Fetal biometry, umbilical, and uterine artery Doppler ultrasonography were performed. To evaluate the flow in these veins, the pulsatility index, resistance index, and systole/diastole (S/D) ratio were performed.

Results: The mean age of study patients was 25.06 ± 4.36 years and the mean gestational week was 20.03 ± 0.6 weeks. In terms of the umbilical artery pulsatility index (UMBAPI), umbilical artery systolic/diastolic ratio (UMBAS/D),uterine artery resistance index (UARI), uterine artery pulsatility index (UAPI), and uterine artery systolic/diastolic ratio (UAS/D) levels, the mean value of the smoker group was significantly higher compared to the non-smoker group (p<0.001, p=0.043, p=0.021, p=0.020, and p=0.037, respectively). The birth weight of the fetus was significantly lower in the active and passive smoker groups than in the non-smoker group (p=0.009 and p=0.006, respectively). The number of patients diagnosed with intrauterine growth restriction (IUGR) and oligohydramnios were significantly higher in the smoker group than in the passive smoker and non-smoker groups (p=0.003 and p<0.001, respectively). The risk of low birth weight (OR, (95% CI): 3.38 (2.05 - 5.57); p=0.024), oligohydramnios (OR (95% CI): 13.44 (5.22 - 34.57); p=0.001), IUGR (OR (95% CI): 9.33 (4.50 - 19.33); p=0.001), and preterm birth (OR (95% CI): 4.56 (1.25 - 17.32); p=0.001) increased significantly in the active and passive cigarette exposure groups, compared to the non-smokers.

Conclusion: During pregnancy, both smoking and passive exposure to cigarette smoke adversely affect the fetus and the newborn. Uterine and umbilical artery Doppler measurements in pregnant women who smoke are significantly higher than the pregnant women who do not smoke.

## Introduction

Exposure to cigarette smoke affects all stages of human development, including intrauterine life [1]. Maternal smoking is associated with an increased risk of preterm birth, premature membrane rupture, abruptio placentae, placenta previa, low birth weight, and congenital abnormalities during pregnancy [2,3]. Babies whose mothers are exposed to cigarette smoke, even if not active smokers during pregnancy, are at a high risk of low birth weight [3].

Cotinine is the metabolite of nicotine. It was shown to be present both in the amniotic fluid and in the cord blood of the fetus, confirming that it can permeate through the placental barrier [4,5]. Cotinine and nicotine increase the maternal blood pressure and heart rate, while simultaneously decreasing the uterine artery blood flow [5]. Carboxyhemoglobin concentrations of the fetus increase due to the high concentration of carbon dioxide found in cigarette smoke, and decrease the oxygen saturation in the descending aorta and inferior vena cava, eventually causing intrauterine hypoxia and complications during pregnancy [6,7].

In women exposed to cigarette smoke, major vascular changes occur in the uteroplacental bed and, therefore, similar to its utilization in female smokers, Doppler ultrasonography (USG) is the primary diagnostic tool in evaluating the maternal and fetal vascular structures in obstetric complications, such as preeclampsia and uterine development retardation [8].

In the present study, we aimed to investigate the relationship between the changes in the fetal and maternal blood flow during mid-trimester in both active and passive female smokers and obstetric and neonatal complications.

## Materials and methods

This prospective study includes women 20 to 22 weeks of pregnancy with no fetal anomalies, as detected by USG, and with a singleton pregnancy, who were admitted to our obstetrics outpatient clinic in Zekai Tahir Burak Women Research and Training Hospital. The study protocol was approved by the Clinical Research Ethics Committee by approval number 01/05/2009 - 06/05. Informed consent was obtained from each participant. The study was conducted in accordance with the principles of the World Medical Association Declaration of Helsinki. This research was conducted as a residency thesis of Sema Baki, in Zekai Tahir Burak Women Research and Training Hospital.

Sample size

Sample size analysis was performed using the data from Yildiz et al.’s study with 79 pregnant women in the third trimester. The authors evaluated 33 passive smokers, 23 active smokers, and 23 non-smoking control groups in their study. Similar to our study, uterine and umbilical artery pulsatility, and resistance index data were compared between groups. Sample size analysis was performed based on the measurement with the lowest effect size (Umbilical artery plurality index, effect size: 0.254). It was planned to include a minimum of 153 participants in the study, with a minimum of 51 participants in each group (Type 1 error: 0.05; power: 80%; critical F: 3.056; noncentrality parameter λ: 9.87) [9].

Study population

Data were obtained from a total of 196 pregnant women who were admitted to the obstetrics outpatient clinic within the routine screening program. The inclusion criteria of our study were: (i) healthy singleton pregnancy without fetal anomaly in previous follow-ups, (ii) confirmation of singleton pregnancy and absence of fetal anomaly by USG at the index visit, (iii) pregnant women at and after the 20th gestational week. The women who had small for gestational age (SGA), preeclampsia, or spontaneous preterm birth in their previous pregnancy, those who had an underlying medical condition, and those who had two or more miscarriages and any obstetric interventions were excluded from the study. Pregnant women were included in the study sequentially until they reached the target numbers in the sample size analysis. Participants were included in the study with a questionnaire evaluating their sociocultural levels, marital status, and smoking habits. Those with a smoking history of at least 1 to 5 cigarettes per day during mid-trimester were included in the smoker group. Those who were exposed to tobacco smoke at home or in the workplace due to smokers were considered passive smokers, while those who were neither active nor passive smokers were selected as the control group. Age, gravida, parity, number of abortions, date of last period, pregnancy weeks according to USG and date of last period, and habit of cigarette smoking were recorded for all patients. Routine antenatal controls of all participating pregnant women were made prospectively. Starting from their 20th week of pregnancy, the women were examined at least once in each trimester [10]. Fetal and maternal pregnancy results were evaluated. Pregnancy weeks were determined based on the date of the last period and first-trimester USG findings. Newborns born at <37 weeks of gestation were considered preterm, those with a membrane rupture were considered early membrane rupture, newborns born between 37 and 42 weeks of gestation were considered term, and those born at ≥42 weeks of gestation were considered post-term. Those under 2,500 g of birth weight were considered low birth weight, those with a birth weight <10 percentile according to the pregnancy week were considered SGA, and those with <3 percentile were diagnosed with intrauterine growth restriction (IUGR). The detailed neonatal examination was performed immediately after the birth, and the weight and sex of the babies and their Activity, Pulse, Grimace, Appearance, and Respiration (APGAR) scores at 1 and 5 min were recorded.

B-mode and Doppler USG examination

In all pregnant women, USG measurements of biparietal diameter (BPD), femur length (FL), and abdominal circumference (AC) were performed. Doppler USG measurements were performed on the same participants by two different experts (SB and CG) once between 19 weeks 6/7 and 21 weeks 6/7 in the supine position lying slightly toward left. No fetal respiration or movement was allowed during the measurement of all Doppler indices. A minimum of five waves were recorded in each measurement and their mean value was calculated. Special attention was paid to preventing contraction in pregnant women during the measurement. Both uterine arteries at the medial section of the iliac vein and at the level of isthmus were visualized, and uterine artery Doppler USG measurement was recorded and their mean value was calculated. For Doppler USG of the umbilical artery, localization and structure of the chord were analyzed. The measurements were performed far from the fetus and placenta from the initiation angle mostly kept under 50 degrees. To evaluate the flow in these veins, the pulsatility index (PI), resistivity index (RI), and systole/diastole (S/D) ratio were performed. Fetal biometry, umbilical, and uterine artery Doppler USG were performed using the SIEMENS ACUSON x150 CH Color Doppler device (Siemens Healthcare, Erlangen, Germany) with a single applicator and a 2 to 5 MHz convex probe. In our study, inter-class correlation coefficient (ICCC) values were calculated for each of the nine USG measurements obtained to evaluate the agreement of the sonographers. The lowest ICCC value obtained was 0.79 (p=0.023), while the highest ICCC value was 0.93 (p<0.001).

Statistical analysis

Statistical analysis was performed using the SPSS for Windows version 24.1 (SPSS Inc., Chicago, IL, USA). Descriptive data were expressed in mean ± standard deviation or median (minimum-maximum), while categorical variables were presented in number and percentage (%). The Shapiro-Wilk normality test was used to analyze the normal distribution of the data. The z-score for each gestational week was derived by using the following formula: z-score = (actual value - mean for the gestational week)/SD (for the gestational week). Median z-score values combining all gestational weeks were then calculated. One-way analysis of variance (ANOVA) was used to analyze the differences in mean values among groups, while the Kruskal-Wallis test was used to analyze the significant differences in median values. If the results of the one-way ANOVA and Kruskal-Wallis test were found significant, post-hoc Tukey’s test or non-parametric multiple comparison tests were used to detect the reasons for the difference. Categorical variables were analyzed using Pearson’s chi-square or Fisher’s exact tests. Multivariate logistic regression analysis was used to examine whether the statistically significant effect of smoking on low birth weight continued after corrections based on gained pregnancy weight, which affects low birth weight and history of low birth weight. The OR and 95% CI were calculated for each variable. ICCC was calculated by reliability analysis. A p value of <0.05 was considered statistically significant.

## Results

The distribution of 196 pregnant women evaluated in our study according to their smoking status was as follows: 50 smokers, 58 passive smokers, and 88 non-smokers. A total of eight women in the passive smoker group and 20 women in the non-smoker group were excluded from the study, as they were lost to follow-up for scheduled antenatal visits. As a result, the distribution of pregnant women evaluated in the analyses was as follows: 50 pregnant women in the smoker group, 50 pregnant women in the passive smoker group, and 68 pregnant women in the non-smoker group.

There was no statistically significant difference in the age, number of abortions, and pregnancy week among active smokers, passive smokers, and non-smokers. Analysis results are presented in Table 1. The parity number in the active smoker group was lower than in the passive smoker and non-smoker groups (p=0.017 and p=0.002, respectively). Body mass index was also lower in the passive smoker group than in the other two groups (p=0.014 and p=0.030, respectively). All participants were married. 42% of the participants were working. The rate of employment in the non-smoker group was significantly higher than in the smoker group (49% and 34%, respectively, p=0.017). The educational status of the participants was categorized as primary, secondary, and higher. It was determined that 31% of all participants were primary, 41% secondary, and 28% with higher education level. The primary education level was significantly higher in the smoker group (46%) compared to the other two groups, while the higher education level was significantly lower in the smoker group compared to the non-smoker group (9%). Secondary education level was similar between groups.

**Table 1:** Demographic Characteristics and Mode of Delivery Data are mean ± SD or number (%) or median (minimum-maximum) as appropriate. NVD: normal vaginal delivery; C/S: cesarean section. The P values in the far right column belong to the ANOVA analysis, the p values in the “a,b,c” explanations are from the post-hoc Tukey test. ^a^Statistical difference between smoker and passive smoker groups (p<0.05). ^b^Statistical difference between smoker and non-smoker groups (p<0.05). ^c^Statistical difference between passive smoker and non-smoker groups (p<0.05).

Variables	Smoker (n=50)	Passive smoker (n=50)	Non-smoker (n=68)	P value
Age	24.4 ± 3.9	24.5 ± 5.2	26.3 ± 4.0	0.061
Parity	0 (0 - 2)^a,b^	1 (0 - 3)^a^	1 (0 - 3)^b^	0.004
Number of abortions	0 (0 - 1)	0 (0 - 1)	0 (0 - 2)	0.784
Body mass index	24.0 ± 3.8^a^	21.7 ± 3.6^a,c^	23.6 ± 4.2^c^	0.009
Gestational week	20.1 ± 0.6	19.9 ± 0.5	20.1 ± 0.7	0.345
NVD	18 (36.0%)	28 (56.0%)	36 (52.9%)	0.092
C/S	32 (64.0%)	22 (44.0%)	32 (47.1%)	0.104

The mean BPD, AC, FL, and umbilical artery RI values were not significantly different among the groups (p>0.05) and are presented in Table 2. In terms of the umbilical artery PI and S/D, the mean value of the smoker group was statistically significantly higher compared to the non-smoker group (p<0.001). In terms of the umbilical artery S/D, uterine artery RI, PI, and S/D SDS levels, the mean value of the smoker group was statistically significantly higher compared to the non-smoker group (p=0.045, p=0.019, p=0.014, and p=0.028, respectively).

**Table 2:** Comparison of Standard Deviation Scores of Fetal Measurement and Doppler Parameters for Each Group AC: abdominal circumference; BPD: biparietal diameter; FL: femur length; UMBARI: umbilical artery resistance index; UMBAPI: umbilical artery pulsatility index; UMBAS/D: umbilical artery systolic/diastolic ratio; UARI: uterine artery resistance index; UAPI: uterine artery pulsatility index; UAS/D: uterine artery systolic/diastolic ratio. Data are mean ± SD or number (%) or median (minimum-maximum) as appropriate. Data were expressed as “z-scores”. The P values in the far right column belong to the ANOVA analysis, and the p values in the “a,b,c” explanations are from the post-hoc Tukey test. ^a^Statistical difference between smoker and passive smoker groups (p<0.05). ^b^Statistical difference between smoker and non-smoker groups (p<0.05). ^c^Statistical difference between passive smoker and non-smoker groups (p<0.05).

Variables	Smoker (n=50)	Passive smoker (n=50)	Non-smoker (n=68)	P value
BPD	-0.17 ± 1.11	0.05 ± 1.19	0.09 ± 0.71	0.363
AC	-0.11 ± 1.07	-0.14 ± 1.08	0.18 ± 0.84	0.143
FL	-0.19 ± 1.05	0.06 ± 1.20	0.09 ± 0.74	0.265
UMBARI	0.26 ± 0.98	-0.04 ± 0.86	-0.16 ± 1.06	0.072
UMBAPI	0.39 ± 1.19^a^	0.05 ± 0.99	-0.32 ± 0.70^a^	<0.001
UMBAS/D	0.29 ± 1.08^b^	-0.09 ± 0.88	-0.15 ± 0.97^b^	0.043
UARI	0.26 ± 1.08^b^	0.07 ± 0.79	-0.24 ± 1.02^b^	0.021
UAPI	0.30 ± 1.07^b^	0.00 ± 0.77	-0.22 ± 1.04^b^	0.020
UAS/D	0.27 ± 1.10^b^	0.00 ± 0.76	-0.20 ± 1.03^b^	0.037

The birth weight of the fetus was significantly lower in the active and passive smoker groups than in the non-smoker group (p=0.009 and p=0.006, respectively). However, no significant difference was found in the birth week among the three groups. In the smoker group, the APGAR scores at 1 and 5 min were lower, indicating a significant difference compared to the other groups (p=0.046 and p=0.017, respectively).

In addition, there was no significant difference in the mode of delivery among the three groups. The obstetric complication results of the study patients are presented in Table 3. However, the preterm birth rate was significantly higher in the passive smoker group than in the smoker and non-smoker groups (p=0.003 and p=0.008, respectively). The number of patients diagnosed with IUGR was significantly higher in the smoker group than in the passive smoker and non-smoker groups (p=0.003 and p<0.001, respectively). The number of patients diagnosed with oligohydramnios was significantly higher in the smoker group than in the passive smoker and non-smoker groups (p=0.003 and p<0.001, respectively). The rate of early membrane rupture was significantly higher in the smoker and non-smoker groups than in the passive smoker group (p<0.001 and p<0.001, respectively). Although the risk of low birth weight increased in the smokers, compared to the non-smokers, this result was not statistically significant (p=0.073).

**Table 3:** Obstetric Complications of Study Groups EMR: early membrane rupture; IUGR: intrauterine growth restriction. ^a^Statistical difference between smoker and passive smoker groups (p<0.05). ^b^Statistical difference between passive smoker and non-smoker groups (p<0.05). ^c^Statistical difference between smoker and non-smoker groups (p<0.05).

Variables	Smoker (n=50)	Passive smoker (n=50)	Non-smoker (n=68)	P value
Preterm birth	0 (0%)^a^	9 (18.0%)^a,b^	2 (2.9%)^b^	<0.001
IUGR	14 (28.0%)^a,c^	3 (6.0%)^a^	2 (2.9%)^c^	<0.001
Oligohydramnios	16 (32.0%)^a,c^	4 (8.0%)^a^	2 (2.9%)^c^	<0.001
EMR	12 (24.0%)^a^	0 (0%)^a,b^	16 (23.5%)^b^	<0.001

The analysis results on the effect of active and passive cigarette exposure on adverse perinatal outcomes are presented in Table 4. The risk of low birth weight increased statistically significantly in the active and passive cigarette exposure groups, compared to the non-smokers (OR (95% CI): 3.38 (2.05 - 5.57); p=0.024). The risk of oligohydramnios increased statistically significantly in the active and passive cigarette exposure groups, compared to the non-smokers (OR (95% CI): 13.44 (5.22 - 34.57); p=0.001). The risk of IUGR increased statistically significantly in the active and passive cigarette exposure groups, compared to the non-smokers (OR (95% CI): 9.33 (4.50 - 19.33); p=0.001). The risk of preterm birth increased statistically significantly in the active and passive cigarette exposure groups, compared to the non-smokers (OR (95% CI): 4.56 (1.25 - 17.32); p=0.001).

**Table 4:** Effect of Cigarette on Adverse Perinatal Outcome EMR: early membrane rupture; IUGR: intrauterine growth restriction ^a^Statistical difference between smoker and passive smoker groups (p<0.05). ^b^Statistical difference between passive smoker and non-smoker groups (p<0.05). ^c^Statistical difference between smoker and non-smoker groups (p<0.05).

Variables	Smoker (n=50)	Passive smoker (n=50)	Non-smoker (n=68)	Odds ratio (95% Confidence Interval)	P value
Low birth weight (<2,500 g)	6 (12.0%)	8 (16.0%)^c^	2 (2.9%)	3.38 (2.05 - 5.57)	0.024
Oligohydramnios	16 (32.0%)^a.c^	4 (8.0%)^a^	2 (2.9%)^c^	13.44 (5.22 - 34.57)	0.001
EMR	12 (24.0%)^a^	0 (0%)^a,b^	16 (23.5%)^b^	1.28 (0.81 - 2.00)	0.074
IUGR	14 (28.0%)^a,c^	3 (6.0%)^a^	2 (2.9%)^c^	9.33 (4.50 - 19.33)	0.001
Preterm Birth	0 (0%)^a^	9 (18.0%)^a,b^	2 (2.9%)^b^	4.56 (1.25 - 17.32)	0.001

## Discussion

We evaluated the effects of maternal active and passive smoking exposure on uterine and umbilical arterial blood flow and obstetric-fetal adverse outcomes. The most prominent results of our study: (i) it was determined that uterine artery blood flow was significantly higher in the active smoking pregnant group compared to the non-smoking pregnant women, (ii) IUGR and oligohydramnios were significantly higher in the active smokers’ group compared to the other groups, (iii) active and passive smoking exposure is associated with poor obstetric and fetal outcomes in pregnant women, (iv) smoking exposure is a risk factor for fetal and obstetric adverse outcomes.

Numerous studies are showing that smoking is associated with increased complications during pregnancy [2,3,11-16]. According to these studies, the risk of detached placenta increases by 1.4 to 4-fold, preterm birth by two-fold, early membrane rupture by two-fold, and sudden infant death by two-fold in pregnant women who smoke [2,11-14]. In addition, there are strong pieces of evidence of the role of smoking in low birth weight in recent years [15,16].

Passive smoking is another issue that is as harmful as active smoking. One-third of pregnant women are unintentionally exposed to cigarette smoke [17]. Several studies have indicated that passive smoking or exposure to environmental tobacco smoke (ETS) can lead to adverse birth outcomes, such as low birth weight, IUGR, and early pregnancy loss [18]. In this study which we investigated the effects of active and passive smoking on fetoplacental blood flow and their relationship with obstetric and neonatal complications, we found that parity decreased, umbilical and uterine artery resistance increased, oligohydramnios and intrauterine development retardation were more common, and babies had lower birth weight than those with non-smoker mothers in women who were active smokers, and that preterm birth frequency increased in passive smokers.

The effect of cigarette smoking on fetal birth weight is mediated primarily via exposure to noxious substances, such as carbon monoxide and nicotine, which can cross the placenta and interfere with fetal oxygenation [19]. In our study, consistent with the literature, there was a statistically significant difference in the birth weight among the groups, and the birth weight was lower in active and passive smoker groups than in the non-smoker group [15].

Uterine artery hemodynamic disturbances, including increased resistance and decreased blood flow characterized by increased uterine artery S/D, RI, and PI have been reported in the case of acute or chronic maternal smoking [20,21]. These results are consistent with our findings. In addition, maternal smoking was reported to be associated with increased resistance in the umbilical artery blood flow, which was represented by an increased umbilical artery S/D, PI, and RI [20,22]. In the present study, we observed that umbilical artery S/D and PI increased in the active smoker group, whereas umbilical artery RI was similar in all groups. On the other hand, Newnham et al. and Pringle et al. found no pathological findings in the mid-trimester uterine and umbilical artery Doppler USG studies [23,24]. The results we obtained support the results obtained in the other studies we discussed. However, one of the points where our study makes a difference is the evaluation of active and passive smokers together. With this evaluation, it was tried to emphasize the obstetric and fetal negative effects of cigarette smoke exposure. In addition, in our study, Doppler measurements were standardized with z-score analysis. Unlike their counterparts, the deviations of these Doppler measurements from normal were tried to be expressed more clearly.

Irrespective of the underlying mechanism, smoking indisputably adversely affects fetal growth and development, as suggested by previous studies [16,25]. Besides its role in the development of complications during pregnancy, it causes low birth weight, intrauterine development retardation, and preterm birth, thereby, substantially increasing neonatal mortality and morbidity rates and associated expenses [12,15].

There are several limitations to this study, first of all, this study is not randomized. The other limitation is it reflects the experience in just one tertiary care hospital and participants were not classified according to smoking status characterized as light, moderate, or heavy smokers based on the number of cigarettes smoked per day.

## Conclusions

In conclusion, during pregnancy, both smoking and passive exposure to cigarette smoke adversely affect the fetus and the newborn. Uterine and umbilical artery Doppler measurements in pregnant women who smoke are significantly higher than the pregnant women who do not smoke. Cigarette smoke exposure is associated with adverse fetal and obstetric outcomes and increases the risk of these adverse outcomes.
